# Crusted scabies mimicking psoriasis in a patient with type 1 diabetes mellitus^☆^^☆☆^

**DOI:** 10.1016/j.abd.2020.06.020

**Published:** 2021-03-20

**Authors:** Yuanyuan Wang, Yeqiang Liu, Fu-Quan Long

**Affiliations:** a Department of Sexually Transmitted Disease, Shanghai Skin Disease Hospital, Tongji University School of Medicine, Shanghai, China; b Department of Pathology, Shanghai Skin Disease Hospital, Tongji University School of Medicine, Shanghai, China

Dear Editor,

Crusted or Norwegian scabies is an uncommon but highly contagious form of scabies, which is caused by the mite *Sarcoptes scabiei* var. *homini*, and mainly occurs in immunocompromised populations or people with poor sanitary conditions.^1^ Diagnosis can still be challenging, since the crusted lesions can imitate many kinds of skin conditions, such as psoriasis. We herein report a case of a diabetic patient with crusted scabies mimicking psoriasis vulgaris which had been misdiagnosed for 2-years.

A 62-year-old man who complained of scaled plaques on the glans and buttocks for 2-years was referred to our clinic. Two years ago, the patient noticed some red papules on his glans and buttocks with an itching sensation, and the lesions gradually fused as plaques with white scales. He had been multiply diagnosed with psoriasis vulgaris and prescribed topical steroid treatment such as triamcinolone for a long time without significant improvement. The patient has a medical history of type 1 diabetes mellitus for more than 15-years but has never been well controlled.

Physical examination revealed erythematous plaques covered with thick white scales on the glans and gluteal region (Fig. 1), and small amount of scattered erythematous papules around the trunk and genitalia. Blood work-up showed a significantly elevated level of fasting blood glucose at 18.4 mmoL/L. The serological test of *Treponema pallidum* particle agglutination and HIV were both negative. A biopsy from his glans lesions revealed numerous mites and eggs transects in the stratum corneum (Fig. 2). A diagnosis of crusted scabies was confirmed. After treatment with crotamiton 10% cream once a week for two weeks, the lesions started to diminish. At his fourth week's follow-up, the skin lesions disappeared (Fig. 3).

**Figure 1:**
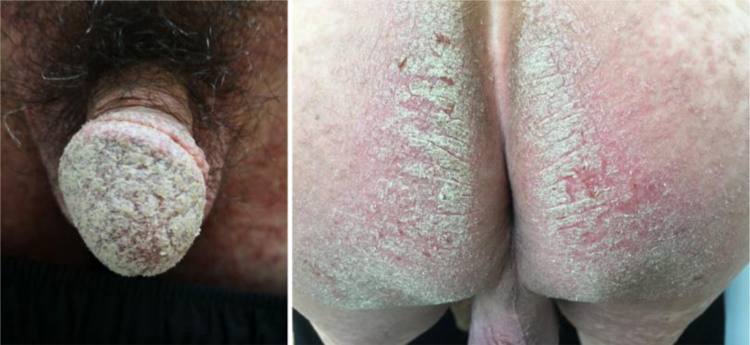
Erythematous plaques covered with thick white scales on the glans and gluteal region.

**Figure 2:**
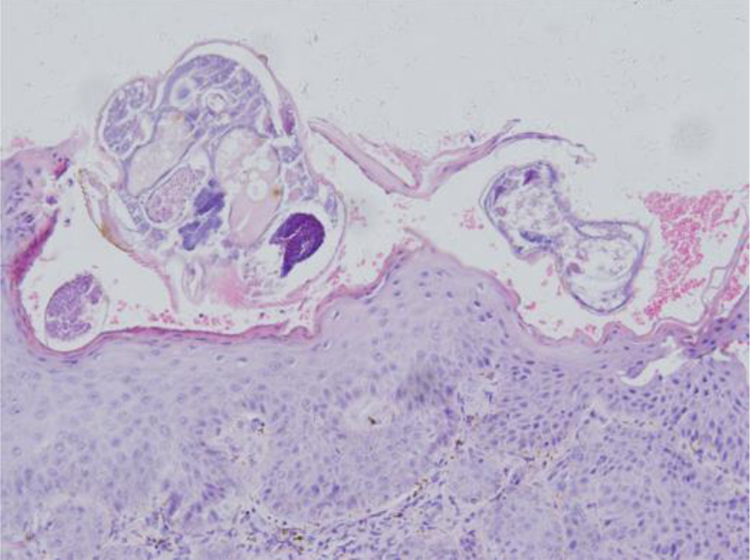
Skin biopsy from glans showed hyperkeratosis, acanthosis of stratum malpighii. Stratum corneum revealed multiple subcorneal burrows containing *Sarcoptes scabiei* larvae and eggs. Dermis had inflammatory cell infiltration rich in eosinophils and lymphocytes (Hematoxylin & eosin stain, original magnification, ×20).

**Figure 3:**
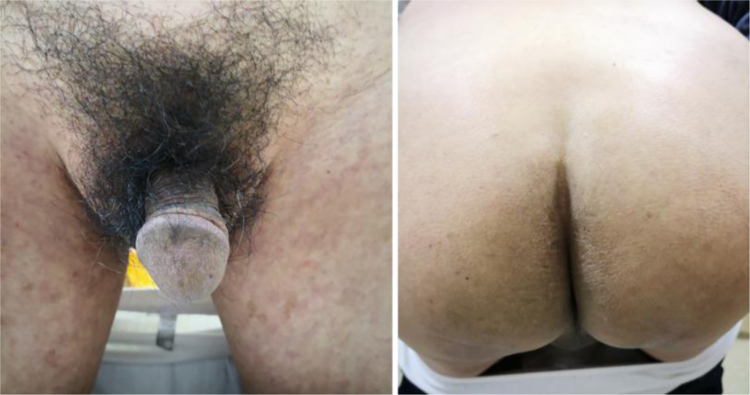
At the fourth week’s follow-up, the skin lesions almost disappeared.

Previous studies have shown that several pre-existing conditions may predispose these crusted scabies, such as AIDS, T-cell lymphoma, Down syndrome and immunosuppression in transplant recipients.^1^, ^2^ In such conditions, the host’s immune system is overwhelmed and inadequate to defend against the parasitic mites, resulting in its subsequent, unrestrained growth. Diabetes mellitus was also one common comorbidity in patients with scabies. Some studies had revealed that elderly diabetics exhibited marginally higher risks of scabies, especially in the patients with type 1 diabetes mellitus.^3^

Crusted scabies is characterized by massive colonization of mites in the epidermis, which lead to keratinocytic hyperplasia, crusted or superimposed eruptions. Psoriasis is one of the important differential diagnosis. Interestingly, it was reported that patients with scabies have a higher risk of subsequent psoriasis.^4^ It is hard to interpret this association, but the common immunopathology involving the T-helper 17 cell-mediated inflammatory pathway may contribute to it.^4^ Based on this fact, physicians may consider implementing assessments of psoriatic symptoms in patients with scabies.

Inappropriate treatment can lead to the worsening of lesions. Using topical corticosteroids can decrease pro-inflammatory cytokines and phagocyte activity, which can thus alter the natural course of scabies, which lead to reduced immune responses and an itching sensation and then promote a crusted scabies appearance.^5^ In our patient, the duration of misdiagnosis lasted for 2-years, mainly due to the immunosuppressive effects of repeated topical corticosteroids in addition to diabetes mellitus.

## Financial support

None declared.

## Authors’ contributions

Yuanyuan Wang: Approval of the final version of the manuscript; elaboration and writing of the manuscript; effective participation in research orientation; critical review of the literature.

Yeqiang Liu: Approval of the final version of the manuscript; effective participation in research orientation; intellectual participation in pathology research of the case; critical review of the manuscript.

Fu-Quan Long: Fu-Quan Long: Approval of the final version of the manuscript; conception and planning of the study; critical review of the literature and manuscript; elaboration and writing of the manuscript, and in charge of the whole manuscript.

## Conflicts of interest

None declared.
